# Chrono-immunotherapy’s current and future optimization strategies: immunotherapy timing in line with the circadian rhythm brings longer survival benefits

**DOI:** 10.3389/fimmu.2026.1777437

**Published:** 2026-02-16

**Authors:** Daiwei Liu, Zhanlin Li, Huijuan Cui, Hua Zhang, Hai Li, Xiaoyuan Wu

**Affiliations:** 1 Department of Traditional Chinese Medicine, Organization The First Affiliated Hospital of Hebei North University, Zhangjiakou, China; 2 Department of Hebei Key Laboratory of Pathogenic Mechanisms and Diagnosis & Treatment Technologies for Lung Microbiome, Organization The First Affiliated Hospital of Hebei North University, Zhangjiakou, China; 3 Department of Integrated Traditional Chinese and Western Medicine Oncology, China-Japan Friendship Hospital, Beijing, China; 4 Department of Respiratory Internal Medicine III, Zhangjiakou First Hospital, Zhangjiakou, China; 5 Department of Oncology, Zhangjiakou First Hospital, Zhangjiakou, China

**Keywords:** chrono-immunotherapy, efficacy, immunotherapy, the circadian rhythm, tumors

## Abstract

Immune checkpoint inhibitors (ICIs) have revolutionized the treatment landscape for malignant tumors such as advanced lung cancer, but their efficacy is limited. In recent years, the circadian rhythm has provided a brand-new optimization strategy for immunotherapy. This perspective article aims to explore the potential mechanisms of the interaction between immunotherapy and circadian rhythms, reviews clinical evidence supporting that “time-of-day receipt of ICIs brings better therapeutic effects”, and conduct an in-depth analysis of the current practical challenges of chrono-immunotherapy. And emphasize the potential of “chrono-immunotherapy” as a valuable therapeutic approach.

## 1 Introduction

We are currently witnessing a transformation in cancer treatment due to the advent of immunotherapy (1). Global cancer statistics indicate that the incidence and mortality rates of lung cancer remain alarmingly high, presenting a substantial public health challenge (2). Immune checkpoint inhibitors (ICIs), particularly those targeting the PD-1/PD-L1 pathway,have emerged as the standard first-line treatment for lung cancer patients without driver mutations.Nevertheless, clinicians and researchers must confront a pressing issue:despite the promise of immunotherapy for long-term survival, response rates remain limited, and both primary and secondary resistance are common (3). Furthermore, the efficacy of these treatments appears to have reached a plateau. We urgently need to precisely screen out the beneficiaries and maximize their therapeutic effects.

In this context, we have focused that the circadian rhythm system serves as the body’s endogenous time regulator. From microorganisms to humans, almost all living beings have evolved biological clocks with a cycle of approximately 24 hours, enabling them to predict and adapt to environmental changes between day and night (4). Increasing evidence suggests that nearly every facet of the immune system (5), including lymphocyte development, migration, and effector function, is intricately regulated by core clock genes (6). The administration time may be an important factor in determining the efficacy and toxicity of anti-cancer drugs (7). According to the latest assessment from CANCERS discovery (8), aligning the timing of ICIs administration with the body’s inherent circadian rhythm may be the key to breaking through the current efficacy limitations (9). Therefore, we propose the new research direction of chrono-immunotherapy, which focus the role of chronotherapy in ICIs efficacy.

## 2 Mechanism

The exploration of pathophysiological basis of the immune circadian rhythm can initially clarify the mechanism by which the timing of drug administration affects the efficacy of immunotherapy (10). This is not a simple summary that “immunity is stronger in the morning”, but rather involves a complex and coordinated process of immune cell recruitment and activation regulated by the circadian rhythm (11).

At the molecular level, core clock genes (CLOCK, BMAL1, Per, Cry) function independently within nearly all immune cells via transcription-translation feedback loops (12, 13). These clock genes regulate cellular metabolism and functional status (14), while also directly overseeing essential molecules involved in the immune response (15). At the same time, immune cells themselves can also express core clock genes such as BMAL1 (16). Research conducted by Xinyue Guo et al. demonstrated that morning administration could upregulate the expression of BMAL1 and PER2 in mice while inhibiting PD-L1, which significantly reduced tumor burden (9).

Studies have demonstrated that the migration, activation and differentiation of T cells (17, 18), along with the expression of the costimulatory molecule CD28, exhibit significant diurnal variations (19). The uptake, processing and presentation of antigens by dendritic cells(DCs) (20) are regulated by the biological clock (21). The timing of DCs migration to lymph nodes aligns with the peak period of T cell response (22), thereby enhancing the immune response (23). Administering ICIs when the immune system is most “alert” can alleviate T cell inhibition and elicit a more robust and sustained anti-tumor response (24).

Moreover, the circadian rhythm of the tumor microenvironment plays a significant role. Tumor-associated macrophages(TAM) (25) and myeloid-derived suppressor cells(MDSCs)that express PD-L1 exhibit alignment with the circadian rhythm (26). Additionally,tumor vascular permeability (10), cytokines levels (27), gut microbiota composition (28), and the expression of immunosuppressive receptors (29) demonstrate periodic fluctuations. These dynamics result in increased immune cell infiltration during the daytime period (30), facilitating a transition of the tumor microenvironment from an immunosuppressed state to one that permits immune activity (30), thereby enhancing the therapeutic efficacy of ICIs.

Cutting-edge research has elucidated the mechanisms underlying the influence of administration timing.During the sleep period of mice (31), which corresponds to nighttime in humans,macrophage activity in the sinus cavity of lymphoid organs is at its peak for phagocytosing and clearing antibodies.Consequently, ICIs administered in the afternoon or evening are more likely to be non-specifically eliminated by these cells (32), resulting in a diminished effective drug concentration at the tumor site and reduced binding to T cells (33). In contrast, early morning infusion of ICIs occurs when the inhibitory function of regulatory T cells (Tregs) in peripheral blood is relatively low. This timing enhances the T-cell-mediated immune response and promotes T-cell receptor (TCR) production, thereby strengthening the anti-tumor effect.

Collectively, nearly all aspects of the immune response, encompassing both innate and adaptive mechanisms, exhibit circadian rhythms (34). The efficacy of immunotherapy relies on the precise orchestration of a series of events: antigen presentation (35), T cell activation, migration, infiltration, and cytotoxic activity (36). Each component of this sequence adheres to a circadian rhythm. Therefore, the intervention of ICIs therapy at the appropriate circadian time could theoretically enhance both the intensity and quality of the immune response (37).

## 3 Current situation

We included the evidence from retrospective studies and randomized controlled trials (RCTs) on the relationship between the efficacy of ICIs and the duration of administration.

Multiple retrospective studies have provided preliminary butevidence yet that remains to be validated.The earliest investigation demonstrated that the administration of immune checkpoint inhibitors at specific time points significantly influenced the overall survival rate of patients with advanced melanoma (38), indicating the potential of chronotherapy. Multiple analyses of advanced NSCLC patients undergoing ICIs monotherapy consistently revealed that those treated in the morning (typically defined as before 11:30) exhibited statistically significant improvements in objective response rate (ORR), progression-free survival (PFS), and overall survival (OS) compared to patients treated in the afternoon (39). This correlation has been observed across various types of ICIs (anti-PD-1/PD-L1) and different cancer types, including metastatic melanoma (40), lung cancer (41), kidney cancer (42), urothelial cancer (43), Head and neck cancer (44), esophageal cancer (45), gastric cancer (46), and liver cancer (47), suggesting the possibility of a universal phenomenon (37). These real-world research studys have, for the first time, closely linked the circadian rhythm theory in the laboratory to the long-term survival outcomes of patients.

Retrospective studies have limitations due to potential influences on the timing of administration,such as clinical workflow, highlighting the necessity for prospective randomized controlled trials (RCTs) to confirm this hypothesis. Currently, several phase I and II RCTs are underway to systematically compare the efficacy of ICIs when administered in the morning versus the afternoon in **Table 1**. These trials have implemented a standardized dosing schedule and incorporated analysis of biomarkers and core clock genes.They initially explored and found that the CD8+T cell activity was better in the early-stage dosing group (48), aiming to elucidate differences in efficacy and potential molecular mechanisms.While the results of these prospective studies are pending, the commencement of such investigations signifies a transition in cancer treatment emphasis from focusing solely on “what to administer” to also considering “when to administer.

**Table 1:** Multiple phase I and II RCTs are ongoing to systematically compare the impact of morning versus afternoon administration on ICIs efficacy.

Identifier	Study type	Agent	Tumor type	Grouping conditions	Outcome measures	Recruitment status
NCT07155317	II trial	Ipilimumab and Nivolumab	Unresectable Melanoma	8:00-11:00;11:00-14:00;14:00-17:00	PFS;AEs、MSS、OS、QOL	Not yet recruiting
NCT06882174	I trial	Pembrolizumab	NSCLC	8:00-10:00;10:00-12;00、12:01-14:00、14:01-17:00	PFS	Not yet recruiting
NCT06294678	I trial	Stem Cell	Hematological Malignancies	11:30-12:30;17:30-18:30	The incidence of grade II to IV aGVHD	Recruiting
NCT05549037	I trial	Chemotherapy plus pembrolizumab or sitilimab	NSCLC	before 15:00;after 15:00	PFS;ORR、OS、DoR	Recruiting
NCT04122456	I trial	Exposed to UVB radiation	Day or night shift workers	8:00;16:00	Level of the DNA repair factor XPA;core clock genes	Recruiting

Significant differences exist in the time threshold (ToD) settings across the aforementioned studies. Various studies have employed distinct time thresholds to delineate “morning” and “afternoon,” ranging from 11:30 a.m. to 4:30 p.m. The fundamental methodological issue arises from the application of a uniform social clock time to segment the administration period, thereby neglecting individual variations in biological clock times (49). This discrepancy in the definition of TOD represents a systematic measurement error (50). In epidemiological studies, it often results in the dilution of effect estimates, complicating the replication and direct comparison of results (51). This issue significantly impedes meta-analysis and the exploration of underlying mechanisms. Consequently, future research should meticulously document administration times and gather individual circadian covariates to facilitate the transition from descriptive phenomena to precise immunotherapy informed by the biological clock.

## 4 Challenge

Although clinical trials have provided strong evidence, the translation of “Chrono-immunotherapy” into clinical practice faces many significant challenges.

Medical institutions typically manage a substantial volume of patients seeking treatment (52) and the infusion appointment system is inherently complex (53). Requiring all patients to receive treatment within the morning time window would disrupt the existing framework for medical resource allocation and could result in delayed care for some individuals. Although patients may be admitted to the hospital one day in advance, scheduling treatment for the following day would inevitably impose a substantial burden on those living in remote areas or those with limited mobility (8).

Many workflows of hospitals and clinics has been formed through decades of practice. Modifying administration times necessitates the reorganization of various components, including nurse scheduling, pharmacy drug preparation, and outpatient procedures (54). This process demands coordinated collaboration hroughout the entire medical system. The costs associated with such changes are substantial, and they may encounter resistance due to systemic inertia.

“Morning” represents a social time, construct rather than a biological one (55). A clear distinction exists between “morning-type individuals” and “night-type individuals” within the population (56). For instance, a “night-type individual” who retires at 2 a.m. and awakens at 10 a.m. may experience peak immune system activity at a time that does not align with the socially defined “morning” (57). Studies have shown that the circadian rhythms of shift workers and individuals who cross multiple time zones are disrupted, leading to imbalances in endocrine factors such as growth hormone, prolactin, thyroid hormone, cortisol, and gonadal steroids (58). This can result in the loss of rhythmic fluctuations in IL-12 and IL-10, thereby affecting the occurrence, progression, and treatment of cancer (59). Sleep dysregulation may elevate levels of pro-inflammatory cytokines (e.g., TNF-α), hindering the effectiveness of ICIs (60) and triggering excessive production of Th1 and Th2 cell-derived cytokines, causing immune system dysfunction (61). These challenges complicate the determination of the optimal universal treatment timing. Temporal variation and circadian rhythm disorders in cancer patients complicate the determination of optimal treatment timing. Research indicates that cancer patients exhibit distinct characteristics, including reduced amplitude of melatonin secretion (62), cancer patients loss of cortisol rhythm (63), and phase disorders in the oscillations of core clock gene expression (64). Furthermore, circadian rhythm disorders are prevalent among patients with advanced cancer and are closely associated with symptom burden, quality of life, disease progression, and survival rates (65, 66). Consequently, the biological clock state of cancer patients markedly differs from that of healthy individuals.This poses significant difficulties for us in defining a universally applicable “optimal dosing time”.

Circadian rhythms not only influence therapeutic efficacy but may also impact the incidence and severity of immune-related adverse events (irAEs). Currently, several studies indicate that the overall incidence of toxicity in the early treatment group is relatively high (67, 68), including skin toxicity and fatigue (69), while no cases of discontinuation of ICIs due to irAEs have been reported (40). Research indicates a gender disparity in the incidence of toxic events, with women experiencing higher rates than men (67). Conversely, some reports suggest that early treatment enhances therapeutic efficacy without leading to a difference in adverse reactions (38, 44). Consequently, it remains uncertain whether administering treatment in morning will concurrently improve efficacy and toxicity.

The relationship between the efficacy and toxicity of ICIs remains an active area of research. Studies have demonstrated a degree of synchrony between these two factors in the context of circadian rhythms (67, 68); however, adverse reactions do not consistently correlate with therapeutic outcomes (38, 44). Current investigations indicate that irAEs are linked to inflammatory responses (70), often characterized by abnormal elevations in cytokines such as IL-6 and TNF-α (71). Inflammation can inhibit the expression of core clock genes (72), upregulate the levels of cytokines, cause circadian rhythm disorders, further aggravate the inflammatory response, and thereby exacerbate irAEs (73). These findings initially suggest that precisely adjusting the administration time of ICIs may enhance the anti-tumor immune response while reducing the risk of irAEs. It is necessary to further verify these hypotheses through large-scale RCTs.

In clinical practice, immune checkpoint inhibitors are frequently administered in conjunction with chemotherapy (74), anti-vascular targeted agents (75), and radiotherapy (76). Circadian rhythms have different effects on each treatment (77–79). Therefore, the selection the optimal timing for administering combine treatments presents significant challenges, highlighting the need for more rigorous trials to validate these approaches.

## 5 Future

**Figure 1:**
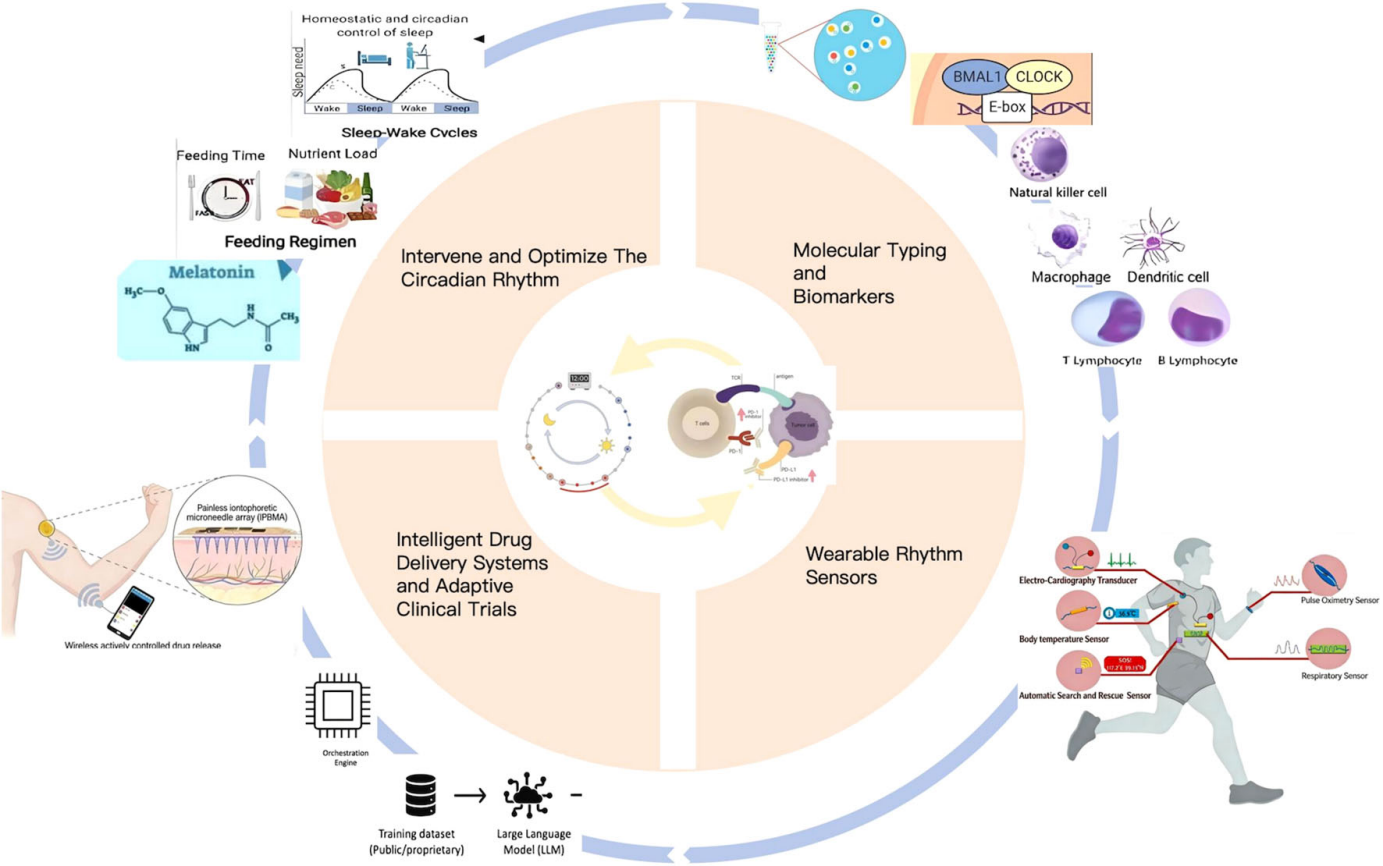
Analyze the characteristic molecular typing and biomarkers of the circadian rhythm of immune cells through single-cell sequencing; Monitor the individualized immune circadian rhythm of patients using wearable rhythm sensors; Develop an intelligent drug delivery systems and adaptive clinical trials to output personalized immune administration times.;By taking multiple measures to intervene and optimize the circadian rhythm of patients, the therapeutic effect of ICIs treatment can ultimately be improved.

In addressing current challenges, our future research direction should not be simply and rigidly scheduling all patients’ treatments in the morning (8). Rather, we should utilize new technologies to precisely screen advantageous patients, provide individualized treatment advantageous times through wearable sensors and intelligent systems, and take multiple measures to actively regulate patients’ circadian rhythms to enhance ICIs efficacy.Further promote the entry of immunotherapy into a highly personalized “chrono-immunotherapy” era (**Figure 1**).

It is essential to identify biomarkers that accurately reflect an individual’s immune rhythmic state. This process involves detecting core clock genes (80)、immune cell subsets (81) and cytokine levels (82) in peripheral blood of patients receiving ICIs treatment at designated time points, identifying key immune cell subsets that are positively or negatively correlated with the efficacy of ICIs,as well as analyzing the rhythmic gene expression profiles of immune cells through single-cell sequencing (83, 84). By combining tumor genomics for fine classification, we may be able to define “circadian rhythmically sensitive” and “circadian rhythmically insensitive” tumors (83), achieving more precise classification at a deeper level.

Based on the above molecular typing, by using utilize genomic (85) and physiological data, including melatonin levels (38), combined with the molecular typing information related to circadian rhythms obtained from single-cell sequencing (86), and through machine learning or deep learning models (87), it can identify the individualized circadian rhythm characteristics of different patients and predict their optimal response time window to ICIs treatment.In the future, key instruments may include smartwatches worn on patients’ wrists or multisensory wearable technology (45, 88). These devices are capable of continuously and non-invasively monitoring rhythm-related physiological parameters, such as rest and activity cycles, heart rate variability, and skin temperature, thereby creating a unique and dynamic “intrinsic biological clock map” for each individual (45). Consequently, treatment decisions will transition from reliance on standard clock time to alignment with the patient’s specific biological rhythm phase (89).

Based on the data from wearable devices (90), intelligent clinical decision support systems can be developed (91). These systems can calculate the specific circadian rhythm phase with the highest immune system activity or the greatest sensitivity of tumor cells to ICIs for each patient (89), and recommend personalized optimal drug delivery Windows (69, 92). Future clinical trial designs are expected to exhibit greater flexibility by adopting “basket” or “umbrella” frameworks (93), which incorporate rhythm states as stratification factors, and allow for dynamic adjustments to treatment plans.

In addition to adjusting the administration times, proactive interventions can optimize the patient’s circadian rhythm to enhance the therapeutic efficacy of ICIs. Research shows that This approach involves intervention measures such as timed light therapy (94), melatonin supplementation (95), time-restricted eating (TRE) (96) and cognitive behavioral therapy (95, 97). These measures can rectify circadian rhythm disorders, improve overall physiological conditions and enhance immune function.By fostering a more favorable internal environment for ICIs treatment, these strategies may ultimately improve therapeutic outcomes for cancer patients undergoing immunotherapy (73).

## 6 Conclusion

Emerging evidence has begun to illuminate the scientific rationale and clinical promise of aligning ICIs administration with circadian rhythm to augment therapeutic efficacy. Our analysis further suggests that morningdosing—,when immune surveillance mechanisms naturally peak —may confer survival benefits in patients with advanced malignancies. This finding that converges with well-documented diurnal oscillations in immune cell dynamics and effector function. A key innovation of this chronotherapeutic approach lies in its paradigm shift from a static, lesion-centric focus to a dynamic, time-aware strategy, thereby establishing “chrono-immunotherapy”as a novel frontier in precision oncology.

While challenges remain,including healthcare system adaptability, interindividual variability in circadian profiles, and the temporal coordination of combination regimens, the “chrono-immunotherapy” represents a compelling avenue to advance toward more dynamic and precise immune-based cancer care. Future RCT studies will be critical to validate optimal timing windows, technological innovations will help critical to validate optimal timing windows, dissect the biological underpinnings of immune circadian rhythm, and targeted circadian interventions will be explored to further enhance immunotherapy outcomes. Ultimately, by synchronizing ICIs delivery with the patient’s endogenous biological clock, we aim to realize a new era of dynamic,personalized cancer immunotherapy.

## Data Availability

The original contributions presented in the study are included in the article/supplementary material. Further inquiries can be directed to the corresponding author.
